# Review of Pharmacotherapy Trialed for Management of the Coronavirus Disease-19

**DOI:** 10.5152/eurasianjmed.2021.20384

**Published:** 2021-06

**Authors:** Kimberly Hall, Fuhbe Mfone, Michael Shallcross, Vikas Pathak

**Affiliations:** 1 Department of Pharmacy, Riverside Health System, Newport News, VA, USA; 2 Department of Internal Medicine, Riverside Health System, Newport News, VA, USA; 3 Department of Family Medicine, Riverside Health System, Newport News, VA, USA; 4 Department of Pulmonary and Critical Care Medicine, Riverside Health System, Newport News, VA, USA

**Keywords:** COVID 19, SARS-CoV-2, Drug therapy, Therapeutics

## Abstract

Since the beginning of the coronavirus disease 2019 (COVID-19) pandemic, there has been substantial progress in the pharmacologic treatment and supportive care of patients hospitalized with active COVID-19 infections. To date there have been numerous medications trialed for COVID-19 management. In this review, our objective is to provide a comprehensive review of the primary literature and clinical applications surrounding some of the prominent drugs and medication classes that have been utilized in those suffering from COVID-19 infections. The medications reviewed in this article include: hydroxychloroquine, remdesivir, azithromycin, dexamethasone, melatonin, tocilizumab, ascorbic acid, and zinc. The medication classes reviewed include: anticoagulation, angiotensin converting enzyme inhibitors, angiotensin II receptor blockers, convalescent plasma, non-steroidal anti-inflammatory drugs, human recombinant soluble ACE2, and the BNT162b2 mRNA COVID-19 vaccine.

## Introduction

The coronavirus study group of the International Committee on Taxonomy of Viruses had proposed the novel coronavirus disease 2019 (COVID-19) infection be categorized as a severe acute respiratory syndrome coronavirus-2 (SARS-CoV-2). This virus has a similar subgenus as severe acute respiratory syndrome (SARS) and is distantly related to the Middle East respiratory syndrome (MERS).^1^ Given the widespread and devastating impact of COVID-19 infections, developing and trialing medications for management and supportive care of COVID-19 infections has been at the forefront of healthcare today. Unfortunately, the hospital management of COVID-19 infections has been based on relatively limited data that is evolving as clinical data emerges.^1^,^2^

In this review article, our objective was to provide a review of the primary literature and clinical applications of some prominent drugs and drug classes utilized for the treatment and support of patients with COVID-19 infections. Details surrounding drug applications are detailed and organized by specific medications below. Medications reviewed in this article, include; hydroxychloroquine, remdesivir, azithromycin, dexamethasone, melatonin, tocilizumab, ascorbic acid, and zinc. In addition, the medication classes reviewed, include anticoagulation, angiotensin converting enzyme (ACE) inhibitors, angiotensin II receptor blockers (ARB), convalescent plasma, non-steroidal anti-inflammatory drugs (NSAIDs), human recombinant soluble ACE2, and the BNT162b2 mRNA COVID-19 vaccine.

## Selection Criteria

We searched PubMed, MEDLINE, and the Cochrane Library using combinations of words that included specific medication names or drug classes with other keywords, such as “COVID-19,” “SARS-CoV-2,” and “coronavirus.” Additional national databases utilized were the World Health Organization and National Institutes of Health. Expert guidance from reputable and established healthcare groups pertaining to COVID-19 management recommendations were assessed throughout this literature review.^1^,^2^

## Hydroxychloroquine/Chloroquine

Chloroquine is a 4-aminoquinoline analog, used as the preferred treatment for malaria worldwide. Hydroxychloroquine has an additional hydroxyl group at the molecule’s end chain and is a disease-modifying antirheumatic drug indicated for the treatment of rheumatoid arthritis, lupus, and porphyria cutanea tarda. Early during the COVID-19 pandemic, it was hypothesized that hydroxychloroquine may prove beneficial in treating COVID-19 owing to its immunomodulatory effects and established reduction of cytokine production to allow an anti-inflammatory response against COVID-19 infections.^2^,^3^

A multi-center, open label, randomized controlled trial (RCT) in China conducted by Tang et al.^4^ treated 75 of 150 hospitalized patients with COVID-19 with hydroxychloroquine. The patients received a loading dose of 1200 mg daily for three days, followed by 800 mg daily for up to three weeks. The primary outcome measured was a probability of negative conversion of severe acute respiratory syndrome. Reportedly, the probability of negative conversion by 28 days in the standard of care versus standard of care plus hydroxychloroquine showed only a 4.1% difference in the treatment arm, but lacked improvement in serial inflammatory markers. However, the hydroxychloroquine group reported some relief in clinical symptoms and improved recovery of lymphopenia.

An observational study of hydroxychloroquine in New York City hospital systems by Geleris et al.^5^ measured intubation or death in patients hospitalized with COVID-19. A total of 1,376 patients who survived for > 24 hours after admission were included in the study; of whom, 811 participants received a 1200 mg loading dose, followed by 400 mg daily for a median of five days. The authors showed no significant association between treatment with hydroxychloroquine and intubation or death, hazard ratio of 1.04 (95% confidence interval [CI] 0.82 to 1.32). Therefore, hydroxychloroquine did not confer a clinically significant reduction in intubation or death.

Another multi-centered, double-blinded RCT by Boulware et al.^6^ have tested the standardized dose of hydroxychloroquine versus a placebo as post-exposure prophylaxis in patients who are moderate to high-risk and asymptomatic. The primary endpoint was incidence of PCR, laboratory-confirmed COVID-19 infection in 821asymptomatic patients. Patients had confirmed household or occupational exposure to COVID-19, with treatment initiated within four days of exposure. Overall, the incidence of infection did not significantly differ in the treatment arm (11.8%) compared with placebo (14.3%), 95% CI 7.0 to 2.2. Side effects were more common in the treatment group (40.1% vs 16.8%, respectively). Thus, post-exposure prophylaxis with hydroxychloroquine may not be effective in preventing infection and may pose more harm than benefit to the patients overall.

In conclusion, additional safety findings have shown reported cases of serious cardiac events and new cases of methemoglobinemia associated with use of high doses. Furthermore, early data suggesting decreased serum viral load and shedding with hydroxychloroquine were not easily replicated in subsequent studies.^6^ Because of the risk for cardiotoxicity, hydroxychloroquine is not recommended for critically ill patients affected by COVID-19.^7^

## Remdesivir

Remdesivir is a nucleoside analog that inhibits viral RNA polymerase and has shown an inhibitory effect on some pathogenic animal and human coronaviruses. The initial place in therapy for remdesivir was reserved for compassionate use in patients with severe COVID-19 infection. This was established on the basis of a multicenter prospective cohort study by Grein et al.^9^ who have reported clinical improvement in patients with severe COVID-19 for compassionate use only. In this study, 53 patients were followed, of which 68% showed an improvement in their oxygenation (N = 36 of 53). Thirty of these patients with severe COVID-19 were intubated (N = 17, 57%) and were successfully extubated. Notably, when applying this data to clinical practice, limitations in interpreting the data should be accounted for owing to lack of standardization or ventilating practices.

As the COVID-19 pandemic became more ubiquitous, the use of remdesivir increased as more studies demonstrated its efficacy. Beigel et al.^10^ completed a randomized, multicenter, double-blinded placebo-controlled trial that evaluated adults hospitalized with COVID-19 and evidence of lower respiratory tract infection. This study enrolled 1,059 patients in which 538 were given remdesivir and 521 were given a placebo. This study demonstrated that after a 10-day course of remdesivir, the median number of days to recovery from COVID-19 infection was approximately 11 days in the remdesivir group compared with 15 days in the placebo group.

Spinner et al.^11^ conducted a randomized multicenter open label trial where 554 patients were assigned to either a five-day course of remdesivir, 10-day course of remdesivir, or standard of care (steroids, hydroxychloroquine, lopinavir-ritonavir, tocilizumab, and/or azithromycin). The patients included were diagnosed with moderate COVID-19 infection as defined by the presence of pulmonary infiltrates and on room air with oxygen saturation greater than 94%. Patients were further classified according to their clinical status on a 7-point scale: Category 1 - death, category 2 - hospitalized receiving invasive mechanical ventilation or ECMO, category 3 - hospitalized receiving noninvasive ventilation or high-flow, category 4 – hospitalized, receiving low-flow supplemental oxygen, category 5 - hospitalized not requiring supplemental oxygen, category 6 - hospitalized not requiring supplemental oxygen or medical care, or category 7 - not hospitalized. Authors demonstrated a 10-day course of remdesivir did not have a statistically significant difference in clinical status compared with the standard of care on the 11^th^ day. However, those receiving a five-day course of remdesivir showed a statistically significant difference in clinical status compared with the standard of care.

These findings were adopted into current clinical practice, with a five-day course of remdesivir becoming standard of care treatment for patients hospitalized with moderate to severe COVID-19.

## Ascorbic Acid

Ascorbic acid is theorized^9^ to support host defenses against infection and may help prevent infection induced oxidative stress. Fowler et al.^13^–^14^ have conducted the CITRIS-ALI trial to determine the effects of IV vitamin C infusion on organ failure scores and biological markers of inflammation and vascular injury in patients with sepsis and ARDS. The CITIS-ALI trial found no significant change in organ failure (SOFA score), inflammatory biomarkers (CRP), or vascular injury (thrombomodulin levels) at 0, 48, 96, and 168 hours in vitamin C arm versus placebo arm. Although evidence in similar infections such as pneumonia and sepsis suggest a possible benefit from vitamin C, there are currently no RCTs examining ascorbic acid efficacy in patients with COVID-19.

## Azithromycin

Azithromycin is a macrolide used for treatment of respiratory, enteric, and genitourinary bacterial infections. Although it is currently used in conjunction with other antimicrobials for the management of COVID-19, there are no established clinical RCTs that support its use. Damle et al.^15^ have examined the antiviral activity of azithromycin with hydroxychloroquine for COVID-19 as a single arm non-randomized study in Marseilles, France. Azithromycin was added to hydroxychloroquine to prevent bacterial superinfection. The findings of this study were that the patients treated with both hydroxychloroquine and azithromycin had a negative PCR nasopharyngeal test for SARS-CoV2 after six days of treatment (N = 6, 100%). In contrast, the comparator group who received hydroxychloroquine monotherapy had a reduced number of patients with negative nasopharyngeal test for SARS-CoV2 after six days (N = 8, 57%). The matching group who received neither hydroxychloroquine nor azithromycin (N = 2, 12.5%) had the lowest number of negative nasopharyngeal tests. The study showed the possible role of azithromycin as a complementary medication to hydroxychloroquine for the reduction of viral load.

Potential mechanisms proposed for azithromycin halting viral replication include blocking endocytosis and the inhibition of viral genetic shedding from lysosomes. Azithromycin is a weak base theorized to raise pH through intracellular accumulation. This pH change prevents viral replication through the inhibition of endosome maturation and function by buffering the acidic environment necessary for removal of the viral capsid resulting in viral replication. *In vitro* studies have compared the degree to which azithromycin reduces this acidic environment and has shown improved inhibition of viral replication over hydroxychloroquine treatment alone.^15^

A retrospective cohort study^16^ examined critically ill patients with confirmed MERS, a virus similar to COVID-19, found azithromycin was not associated with a reduction in 90-day mortality or improvement in MERS-Co-V eradication. In France, an uncontrolled, retrospective analysis of 1,061patients treated with azithromycin and hydroxychloroquine showed a positive clinical outcome and virologic cure in 91.7% of patients treated with both agents within 10 days of treatment.^17^ Conversely, a retrospective multicenter cohort study of confirmed COVID-19 patients in New York City showed no significant reduction in hospital mortality between patients treated with or without azithromycin.^18^

In a multicenter randomized clinical trial published in the Lancet, Furtado et al.^16^ have examined the addition of azithromycin to hydroxychloroquine versus hydroxychloroquine alone in severe COVID-19 infections in Brazil. During this trial, 447 patients were enrolled and given azithromycin plus hydroxychloroquine or hydroxychloroquine alone. This study concluded no significant difference between azithromycin with hydroxychloroquine compared with hydroxychloroquine alone (odds ratio: 1.36, 95% CI: 0.94–1.97, P = .11). This study also showed rates of adverse events such as ventricular arrhythmias, resuscitated cardiac arrest, acute kidney injury, and corrected QT interval prolongation were not statistically significant between groups.

## Dexamethasone

Dexamethasone is a synthetic corticosteroid widely used in the treatment of acute and chronic conditions with anti-inflammatory and immunosuppressive properties. Initial guidance from the US Centers for Disease Prevention and Control (CDC) recommended against the use of systemic corticosteroids in SARS-CoV-2 infections on the basis of evidence from earlier MERS and SARS-CoV-1 viruses. Unlike MERS and previous SARS-CoV-1, COVID-19, especially in the critically ill, presents with an innate immune response transitioning into an adaptive immune response. Those patients with poorer outcomes are found to have higher serum viral loads and localized elevations in viral loads in their lungs, especially at the time of death.^19^,^20^ An RCT by Lee et al.^21^ in Hong Kong, China, measured the effects of systemic corticosteroid treatment on viral loads of SARS-CoV-1virus. This trial compared early initiation of hydrocortisone in critically ill patients with less than seven days of illness with placebo. Early initiation of corticosteroids resulted in subsequently higher serum viral load concentrations in the second and third weeks of illness, delaying viral clearance and possibly poorer outcomes owing to its immunosuppressive response. Preliminary data from other retrospective non-randomized studies in this timeframe demonstrated some clinical benefit.

The RECOVERY Trial^21^ used a controlled, open-label trial of oral and intravenous dexamethasone 6 mg daily for 10 days compared with institution specific care in hospitalized patients with COVID-19 in the United Kingdom. A total of 2,104 patients who received treatment with dexamethasone compared with 4,321 who did not, experienced a significant reduction in the 28-day mortality of 22.9% compared with 25.7%, respectively. For patients who were mechanically ventilated, the dexamethasone group had a decreased 28-day mortality at 23.9% versus 41.4% in the standard care group. The RECOVERY trial identified a shorter duration of hospitalization in patients receiving dexamethasone (median 12 days compared with 13 days) and greater probability of being discharged alive in a 28-day period. Further trials need to be conducted on dosage and timing of dosing to establish a standard treatment regimen that would result in the best clinical outcome in affected patients. For example, the RECOVERY trial dexamethasone dose is half the standard corticosteroid dose for ARDS.^19^,^21^ Nevertheless, steroids in COVID 19 patients as evidenced by the RECOVERY trial and other studies have become standard of care in hospitalized patients with COVID-19.^22^–^25^

## Melatonin

Melatonin is known to have anti-inflammatory properties, free radical scavenging activities, and immunomodulatory functions. It was theorized that melatonin may inhibit tissue damage by causing a decrease in CD147 levels. Notably, SARS-Cov-2 causes tissue damage by increasing the TNF-alpha, MCP-1, and interferon-gamma levels modulated through CD147 cells.^26^ Thus, melatonin may have supportive adjunct activity in treating COVID-19 induced pneumonia, acute lung injury, and acute respiratory distress syndrome through possible CD147 reduction.^27^ Since melatonin production decreases as we age, and the mortality rate in the elderly with COVID-19 remains high, the use of melatonin and its levels were examined in COVID-19 infection.^26^,^27^

Based on higher mortality rates observed in elderly patients infected with COVID-19, low melatonin levels may be a contributing factor to poor clinical outcomes. There are currently many ongoing clinical trials trying to determine the role of melatonin in patients with COVID-19.

## Tocilizumab

Acute respiratory distress syndrome is a devastating complication of COVID-19 infections. Patients who are critically ill with COVID-19 are subject to the development of life-threatening acute systemic inflammatory syndromes characterized by multi-organ failure and cytokine-release syndrome (CRS), marked by elevated levels of the proinflammatory cytokine interleukin 6 (IL-6). In the initial phase of the infection, COVID-19 is thought to antagonize type I INF response in the alveolar epithelial cells of the patient’s airways, leading to rapid viral replication and subsequent activation of numerous inflammatory markers, namely IL-6. This cascade of events causes diffuse alveolar damage within airway epithelium and endothelium causing cellular apoptosis and pulmonary fibrinolysis. This elevation in IL-6 plays a role in the induction of a severe systemic inflammatory response in patients with COVID-19, which leads to the consideration of targeted therapy against this inflammatory marker.^28^,^29^

Tocilizumab is a recombinant humanized anti-IL-6 receptor monoclonal antibody that is indicated for the treatment of autoimmune diseases. However, there is increasing evidence suggesting that tocilizumab may reduce mortality in severe COVID-19 pneumonia and CRS. It is hypothesized that blockade of IL-6 receptor may impact cytokine release storm in patients experiencing a systemic hyper-inflammatory response to COVID-19. A retrospective case-control study^30^ conducted in France examined treatment with tocilizumab for the primary endpoint of death and ICU admissions. Twenty patients were analyzed in the tocilizumab group versus 25 receiving standard therapy, and the result was an overall reduction in ICU admissions and mortality from COVID-19 in the tocilizumab group. Patients with co-morbid conditions (higher Charlson comorbidity index) presented with more severe forms of COVID-19 infection, identified as significant lymphopenia and higher serum CRP levels and subsequently were treated with tocilizumab. However, the tocilizumab group did not result in any ICU admissions, compared with 44% of patients that did not receive tolicizumab. The tocilizumab group experienced death in 25% of patients compared with 48% of untreated patients.^30^ These findings suggested the added benefit of tolicizumab in reducing ICU admissions and overall mortality; however, larger prospective RCTs are required.

Several single-center studies also suggested clinical improvement in patients with COVID-19 receiving tocilizumab who experienced a hyper-inflammatory systemic response associated with ARDS and/or CRS. A 154-patient cohort in an observational controlled study in Michigan studied severe COVID-19 requiring invasive mechanical ventilation and showed that 78 patients receiving tocilizumab experienced a 45% reduction in hazard of death (hazard ratio 0.55). However, patients receiving tocilizumab had a higher rate of super-infection with bacterial pneumonia (54% versus 26%) but did not show a difference in 28-day mortality compared with those without super-infections.^31^ A prospective case series of 100 patients in Brescia, Italy, with COVID-19 and requiring ventilator support, both mechanical and non-invasive, received tocilizumab, two 8 mg/kg infusions 12 hours apart. This study showed overall improvement in the respiratory condition at 10 days from admission, with 77% of the tocilizumab group showing improvement (clearing of bilateral opacities on chest x-ray), and 15 patients successfully discharged to home.^32^ This preliminary data suggests tocilizumab is beneficial in decreasing levels of serum inflammatory markers in critically ill patients. However, standardized dosing, frequency, and number of repeated doses have yet to be determined to ascertain the full benefit of clinical improvement in these patients.

## Zinc

Zinc has been theorized^33^,^34^ to help fight infection by increasing the ability to promote migration of polymorphonuclear cells. A Cochrane review by Singh et al.^35^ have examined five RCTs and concluded that high-dose zinc supplementation in the first 24 hours reduced the duration but not the severity of common cold symptoms compared with placebo. Given the role of zinc in immune function and the theoretical ability to inhibit viral RNA polymerase activity based on *in vitro* studies, zinc is being evaluated in clinical trials for possible efficacy in patients with COVID-19.^35^ Carlucci et al.^36^ have examined the potential benefit of adding zinc to treatment with hydroxychloroquine and azithromycin therapy for 932 patients with COVID-19. The zinc group showed significantly increased frequency of discharge and reduction in mortality or transfer to hospice. However, the addition of zinc did not affect hospital or ICU length of stay nor the duration of mechanical ventilation. Owing to the limited data to support the efficacy of zinc supplementation in COVID-19, no official guidance for or against the use of zinc in patients with COVID-19 exists to date.

## Anticoagulation

Although the true pathophysiologic mechanism of hypercoagulability in patients with COVID-19 is not concretely defined, studies to date have suggested COVID-19 associated coagulopathy may result from an uncontrolled immunothrombotic response to viral infection with COVID-19.^37^–^42^

A retrospective cohort study by Tang et al.^42^ have reported abnormal coagulation patterns and poor prognosis in 183 patients with COVID-19. This study showed coagulation abnormalities such as elevated D-dimers as well as prolonged activated partial thromboplastin time (aPTT) and prothrombin time (PT), which were especially present in those who did not survive. Subsequent studies have supported these coagulation findings in hospitalized patients with COVID-19; the most common abnormalities being elevated D-dimer levels, high fibrinogen levels, minimal prolongation of aPTT and PT, and thrombocytopenia.^43^–^45^

The CHEST Physicians^1^ and American College of Cardiology (ACC) recommend COVID-19 patients should receive prophylactic doses of anticoagulation and do not recommend intermediate or treatment dosed anticoagulation at this time. Notably, these expert panels differed in their preferred anticoagulant. CHEST experts recommend low-molecular weight heparin (LMWH) over unfractionated heparin (UFH) on the basis of no difference in efficacy but to reduce nursing exposure and protective equipment use. Neither panel recommended use of direct acting oral anticoagulants.

Klok et al.^45^ have examined 184 critically ill patients with severe COVID-19 receiving prophylactic anticoagulation. Authors reported a 31% incidence of thrombotic complications in ICU patients despite administration of prophylactic anticoagulation. Additionally, Helms et al.^46^ studied the incidence of thromboembolic complications in critically ill patients with ARDS with and without COVID-19 who received prophylactic (80.7%) or treatment (19.3%) LMWH or UFH. Despite pharmacologic anticoagulation, authors reported a significant increase in thromboembolic events, especially pulmonary embolism in the ARDS COVID-19 group. Both Klok et al.^45^ and Helms et al.^46^ have suggested critically ill patients with COVID-19 may warrant higher doses of anticoagulation than prophylactic doses to prevent negative thromboembolic outcomes. Notably, neither study addressed safety outcomes. Additional studies and clinical practice further support patients with COVID-19 may require higher than prophylactic anticoagulation.^43^–^45^ Given the lack of published randomized control trials examining the efficacy of intermittent and treatment dosed anticoagulation, many expert panels are hesitant to recommend higher doses of anticoagulation in patients with COVID-19. Many clinical trials are ongoing to evaluate safety and efficacy outcomes of various anticoagulation doses in COVID-19.^37^,^39^,^44^,^45^ According to the current evidence, there is insufficient evidence to confirm the use of higher than prophylactic anticoagulation dosing in patients with COVID-19 at this time.

## Angiotensin Converting Enzyme Inhibitors and Angiotensin-II Receptor Blockers

SARS-CoV-2 utilizes angiotensin converting enzyme 2 (ACE2) receptors as an entry mechanism into cells. ACE2 is expressed by epithelial cells of the lung, intestines, kidney, and vasculature. Fang et al.^47^ have detailed how patients with diabetes and hypertension being treated with ACE inhibitors and angiotensin II receptor blockers (ARB) had substantial upregulation of ACE2. This increased prevalence of ACE2 increased their risk of COVID-19 infection. Conversely, there is theoretical benefit of using ACE inhibitors or ARBs to possibly prevent lung damage through inhibiting viral budding of the COVID-19 virus.^47^

To determine if negative outcomes exist between specific anti-hypertensives, Reynolds et al.^48^ evaluated 12,594 patients for theoretical association between ACE inhibitors, ARBs, beta-blockers, calcium-channel blockers, or thiazide diuretics in COVID-19 infections. The authors found no association between the studied antihypertensives and COVID-19. Mancia et al.^49^ further assessed 6,272 patients with COVID-19 to determine if an association existed between use of ACE inhibitors or ARBs with COVID-19 infections. The authors found no association between renin angiotensin aldosterone system (RAAS) blockade with ACE inhibitors and ARBs and COVID-19 infections. Both studies found no correlation between antihypertensive use and severity of COVID-19. These findings are reflected in the current guidelines from the American Heart Association, ACC, Heart Failure Society of America, European Society of Cardiology, and National Institutes of Health (NIH), which recommend the use of ACE inhibitors or ARBs for patients who are already prescribed such agents for cardiovascular disease and do not recommend use for the treatment of COVID-19. At this time, there is no concrete clinical evidence to support the use of ACE inhibitors or ARBs in COVID-19 treatment.

## Convalescent Plasma

Convalescent plasma is collected from patients who have recovered from COVID-19 and which contains polyclonal antibodies thought to provide short-term passive immunity against SARS-Co-V2 virus. Historically, convalescent plasma has been used to treat other viruses with varying degrees of success (H1N1, SARS-1, and MERS).^50^,^51^ Cochrane review by Valk et al.^52^ have assessed whether convalescent plasma or hyperimmune immunoglobulin transfusion was effective and safe for treating 32 patients with COVID-19. The authors concluded very low certainty of evidence to support the effectiveness and safety of convalescent plasma for COVID-19; however, the authors stated a limitation was a high level of variability regarding dose and timing of convalescent plasma administration, donor, and recipient. Liu et al.^53^ have evaluated if administration of convalescent plasma improved supplemental oxygen requirements and survival in 39 patients with COVID-19. This study showed convalescent plasma recipients had improvements in supplemental oxygen requirements by post-transfusion day 14 and improved survival rates in non-intubated patients.

Further evaluation of convalescent plasma on mortality was examined in systematic review of 12 studies.^54^,^55^ The aggregated patient outcomes from randomized-controlled studies showed patients transfused with convalescent plasma exhibited reduced mortality by 13% compared with non-transfused patients with COVID-19. The NIH does not recommend for or against the use of convalescent plasma as trials have notable limitations and bias. Notably, recent data suggests convalescent plasma is more effective if given during the early stages of the COVID-19 infection. However, criteria to receive COVID-19 convalescent plasma and appropriate dosages have not yet been fully evaluated.^50^,^52^,^55^

## Non-Steroidal Anti-Inflammatory Drugs

Fang et al.^47^ speculated a possible link between ibuprofen (NSAID) and COVID-19. A clinical review by Sodhi et al.^56^ have explored ibuprofen as supportive therapy in COVID-19 owing to its possible effect on ACE2 expression. This review added anecdotal evidence to support the speculated link between NSAIDs and negative outcomes in COVID-19.^47^,^56^ Another NSAID drug with theoretical benefit in COVID-19 is indomethacin on the basis of *in vitro* antiviral activity against SARS-CoV-2. Amici et al.^57^ have conducted *in vitro* studies on monkey Vero E6 cells and A72 canine cells, which demonstrated a reduction in viral particle production by blocking viral RNA synthesis. Despite these *in vitro* findings, there is no clinical evidence to support use of indomethacin in COVID-19.

## Human Recombinant Soluble ACE2

Amidst research and treatment efforts for COVID-19, ACE2 has been identified as a cell surface receptor for SARS-CoV2 needed for virus replication. This receptor is a regulator of the RAAS system and protects organ systems and tissues, namely the lungs, from injury. Subsequently, efforts in drug development targeting ACE2 gained traction, and a novel compound synthesized by Apeiron Biologics in Vienna, Austria, has been developed. This compound, the human recombinant soluble ACE2 (hrsACE2), targets two mechanisms of action in COVID-19 treatment. The first mechanism involves targeted suppression of proteins associated with ACE2, which generally spike when viral loads are high. The second mechanism targets an increase in angiotensin II concentrations and RAAS hyperactivity associated with increased viral load. Targeting and suppressing these cascades theoretically minimizes injury to multiple organ systems including the lungs, kidneys, and heart.^58^ The targeting and binding of ACE2 counter-balances the effects of elevated angiotensin II. Up-regulation of ACE2 results in disruption of homeostasis of the renin-angiotensin system and presumed severe lung injury.^58^

A case report recently published by Zoufaly et al.^58^ details a hospitalized 45-year-old female with a seven-day history of prodromal viral symptoms and subsequent diagnosis of COVID-19. She failed standard COVID-19 treatments with worsening clinical presentation and was subsequently administered experimental hrsACE2 therapy. Its administration was scheduled twice daily intravenously for seven days and was generally well-tolerated without any definite side effects.^59^ A decrease in viral load was noticeable after the first day of treatment. A reduction in angiotensin II metabolites and serum cytokine levels (IL-6 and IL-8) commonly associated with cytokine storm in critically ill patients was also documented. Furthermore, inflammatory markers also downtrended with the normalization of angiotensin II levels by day seven.^59^ HrsACE2 for the treatment of COVID-19 appears promising, but is not widely studied yet. Phase 2/3 clinical studies utilizing hrsACE2 are currently ongoing. 58

## BNT162b2 mRNA COVID-19 Vaccine

The BNT162B2 is a nanoparticle-formulated, nucleoside-modified RNA (modRNA) vaccine that encodes a pre-fusion stabilized, membrane anchored SARS-CoV-2 full length spike protein with two Proline mutations that lock the virus in its pre-fusion confirmation. In a multinational placebo-controlled double-blinded efficacy trial, 43,548 participants aged 16 years or older were randomly assigned in the 1:1 ratio to receive the two dose vaccine 21 days apart or placebo. During the study, two 30 mcg doses elicited high SARS-CoV-2 neutralization antibodies and a robust CD8+ and CD4+ T-cell response. This response showed neutralization exceeding the average titer measured in human convalescent serum panels. Subsequently, the authors demonstrated that the two dose regimen of the BNT162b2 was 95% efficacious at preventing COVID-19 infections. Ten cases of severe COVID-19 were reported after the first injection, nine of which were in the placebo group and one in the BNT162b2 group. Amongst the BNT162b2 group, there were eight reported cases of COVID-19 and 162 reported cases of COVID-19 in the placebo group within seven days after the second dose. The efficacy of the vaccine was observed in all enrolled subjects regardless of ethnicity, sex, race, baseline body mass index, and other comorbidities. The BNT162b2 vaccine reactogenicity profile proved safe with participants having reported short-term local injection site pain or minor systemic responses, such as headaches or subjective fevers. The vaccine safety profile coupled with the efficacy data conferring substantial immune response (a 50% neutralization titer after the second vaccine) was the basis for emergency use authorization of the BNT162b2 vaccine during the phase 2/3 trials.

**Main Points**

- Considering the widespread and devastating impact of COVID-19 infections, developing and trialing medications for management and supportive care of COVID-19 infections is at the forefront of healthcare today.
- This article is a comprehensive literature review of inpatient COVID-19 management to include antivirals for direct treatment of the COVID-19 viral infection, supportive agents for side effect management, and other medications or medication classes thought to contribute to the overall treatment.
- The medications reviewed include hydroxychloroquine, remdesivir, azithromycin, dexamethasone, melatonin, tocilizumab, ascorbic acid, and zinc. The medication classes reviewed include anticoagulation, angiotensin converting enzyme inhibitors, angiotensin II receptor blockers, convalescent plasma, non-steroidal anti-inflammatory drugs, human recombinant soluble ACE2, and the BNT162b2 mRNA COVID-19 vaccine.
- Drug specific literature review and evaluation sheds light on possible benefit or lack thereof for each medication or medication class reviewed.
